# Supplementary material for: Architecture, dynamics and biogenesis of GluA3 AMPA glutamate receptors
Source: Nature. 2025 Jul 1;645(8080):535–43. doi: 10.1038/s41586-025-09325-z (PMC12422969; doi:10.1038/s41586-025-09325-z)
Supplement: Supplementary file 1 — Supplementary Figs. 1 and 2 and Supplementary Table 1. Supplementary Fig. 1: uncropped image of the gel presented in Extended Data Fig. 1a. Supplementary Fig. 2: flow cytometry gating strategy. Supplementary Table 1: cryo-EM data collection, refinement and validation statistics. [file 41586_2025_9325_MOESM1_ESM.pdf]

nature portfolio

https://doi.org/10.1038/s41586-025-09325-z

Supplementary information

Architecture, dynamics and biogenesis of GluA3 AMPA glutamate receptors

In the format provided by the authors and unedited

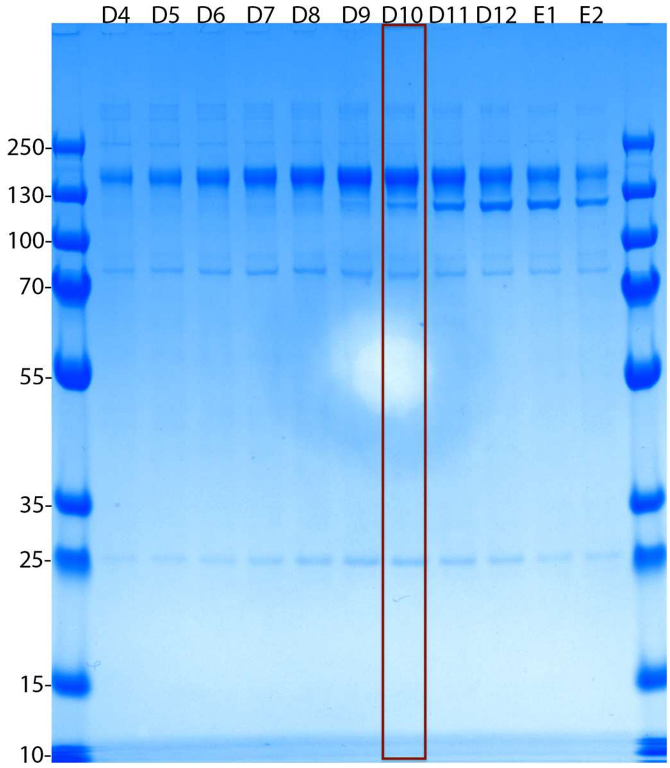

Supplementary Figure 1. Uncropped image of the gel presented in Extended Data Fig. 1a.

Gating was used to include the cell rather than debris. Further gating was not used.

APC was excited by 640nm laser. Signal was collected using channel pass filter 670/14

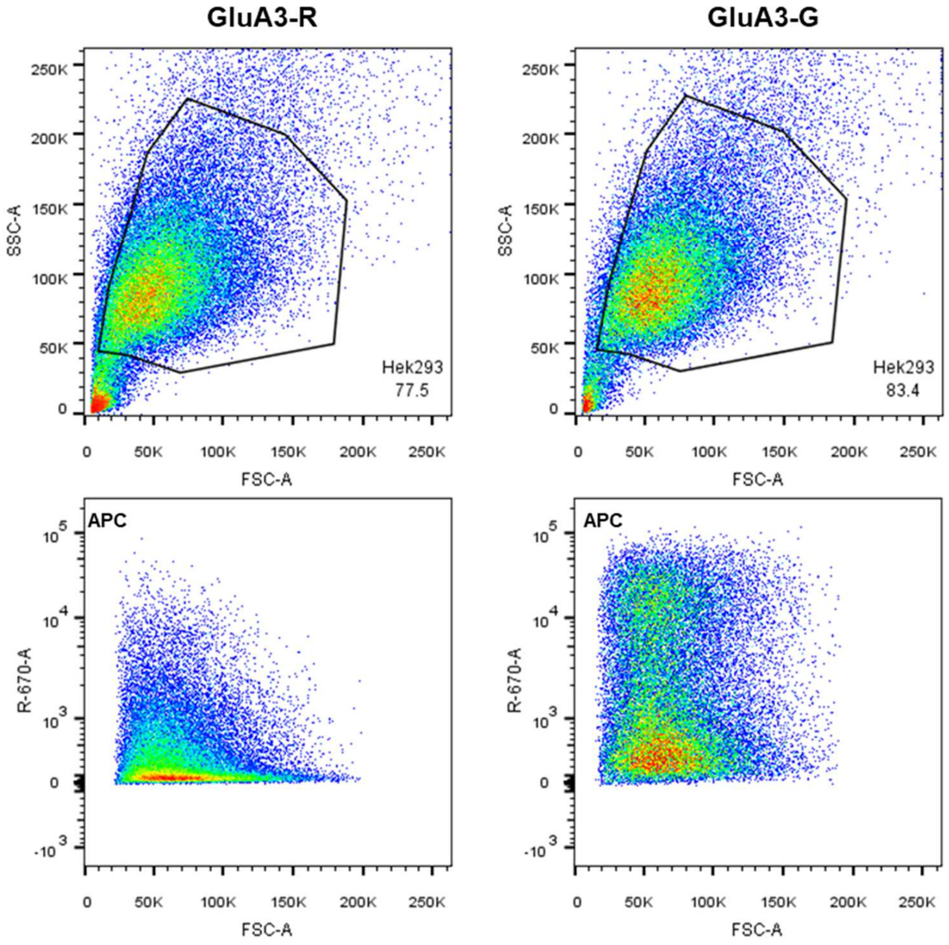

Supplementary Figure 2. Flow cytometry gating strategy.

	Apo state GluA3-G TARP γ2 NTD-LBD tridomain (EMDB-52327) (PDB 9HPE)	Apo state GluA3-G TARP γ2 LBD-TMD tetramer* (EMDB-52325) (PDB 9HPC)	Open state GluA3-G TARP γ2 NTD-LBD tridomain (EMDB-52326) (PDB 9HPD)	Open state GluA3-G TARP γ2 LBD-TMD tetramer* (EMDB-52332) (PDB 9HPK)	Desensitized state GluA3-G TARP γ2 NTD-LBD tridomain (EMDB-52328) (PDB 9HPF)	Apo state GluA3-G (R163I) -TARP γ2 NTD-LBD tridomain (EMDB-52329) (PDB 9HPG)
**Data collection and processing**						
Microscope	TFS Titan Krios	TFS Titan Krios	TFS Titan Krios	TFS Titan Krios	TFS Titan Krios	TFS Titan Krios
Detector	BioQuantum K3+GIF	BioQuantum K3+GIF	BioQuantum K3+GIF	BioQuantum K3+GIF	BioQuantum K3+GIF	Falcon4i+Select risX
Magnification	105,000x	105,000x	105,000x	105,000x	105,000x	130,000x
Voltage (kV)	300	300	300	300	300	300
Electron exposure (e–/Å^2)	38.4-48.2	38.4-48.2	37.6	37.6	36.4	40
Defocus range (μm)	-1.2 to -2.5	-1.2 to -2.5	-1.2 to -2.4	-1.2 to -2.4	-1.2 to -2.5	-1.2 to -2.2
Pixel size (Å)	0.826	0.826	0.826	0.826	0.826	0.955
Symmetry imposed	C1	C1	C1	C1	C1	C1
Initial particle images (no.)	43,313,851	43,313,851	9,055,061	9,055,061	1,795,149	1,258,262
Final particle images (no.)	264,366	1,774,241	104,001	280,853	39,953	151,985
Map resolution (Å)	2.82	2.59	2.96	2.76	3.79	3.35
FSC threshold	0.143	0.143	0.143	0.143	0.143	0.143
Map resolution range (Å)	2.536-9.008	2.246-6.589	2.755-7.746	2.28-6.701	3.38-9.59	2.97-5.16
**Refinement**						
Initial model used (PDB code)	3O21, 5IDE	5IDE, 8CIS	3O21, 3DLN	5IDE, 3DLN, 8CIS	3O21, 4F29	3O21, 5IDE
Model resolution (Å)	2.9	2.7	3.0	3.0	3.7	3.4
FSC threshold	0.143	0.143	0.143	0.143	0.143	0.143
Map sharpening *B* factor (Å^2)	-61.96	-47.56	-61.96	-62.24	-63.28	-72.04
Model composition						
Non-hydrogen atoms	7084	17956	6887	17739	6615	6966
Protein residues	866	2307	863	2257	852	875
Ligands	NAG:4	0	-	-	NAG:2	-
B factors (Å^2)						
Protein	80.79	58.42	104.66	30.62	81.25	38.23
Ligand	112.67	-	-	-	-	-
R.m.s. deviations						
Bond lengths (Å)	0.002	0.003	0.45	0.003	0.002	0.002
Bond angles (°)	0.463	0.449	0.002	0.451	0.478	0.419
Validation						
MolProbity score	1.16	1.06	1.14	1.55	1.67	1.11
Clashscore	3.71	2.72	3.47	8.59	7.37	2.99
Poor rotamers (%)	0.13	0	0.14	0.27	0.00	0.00
Ramachandran plot						
Favored (%)	98.01	99.02	98.23	97.57	96.15	97.91
Allowed (%)	1.99	0.98	1.77	2.33	3.85	2.09
Disallowed (%)	0	0	0	0	0.00	0.00

* The models were initially constructed and refined separately for the locally refined apo state LBD and TMD maps. They were then combined using UCSF ChimeraX and further refined with a focused apo state LBD-TMD map.

Supplementary Table 1. Cryo-EM data collection, refinement and validation statistics
