# Supplementary material for: Architecture, dynamics and biogenesis of GluA3 AMPA glutamate receptors
Source: Nature. 2025 Jul 1;645(8080):535–43. doi: 10.1038/s41586-025-09325-z (PMC12422969; doi:10.1038/s41586-025-09325-z)
Supplement: Supplementary file 2 — Reporting Summary [file 41586_2025_9325_MOESM2_ESM.pdf]

nature portfolio

Corresponding author(s): Ingo H. Greger

Last updated by author(s): Apr 15, 2025

Reporting Summary

Nature Portfolio wishes to improve the reproducibility of the work that we publish. This form provides structure for consistency and transparency in reporting. For further information on Nature Portfolio policies, see our Editorial Policies and the Editorial Policy Checklist.

Statistics

For all statistical analyses, confirm that the following items are present in the figure legend, table legend, main text, or Methods section.

n/a	Confirmed	
☐	☒	The exact sample size (*n*) for each experimental group/condition, given as a discrete number and unit of measurement
☐	☒	A statement on whether measurements were taken from distinct samples or whether the same sample was measured repeatedly
☐	☒	The statistical test(s) used AND whether they are one- or two-sided<br>*Only common tests should be described solely by name; describe more complex techniques in the Methods section.*
☒	☐	A description of all covariates tested
☐	☒	A description of any assumptions or corrections, such as tests of normality and adjustment for multiple comparisons
☐	☒	A full description of the statistical parameters including central tendency (e.g. means) or other basic estimates (e.g. regression coefficient) AND variation (e.g. standard deviation) or associated estimates of uncertainty (e.g. confidence intervals)
☐	☒	For null hypothesis testing, the test statistic (e.g. *F*, *t*, *r*) with confidence intervals, effect sizes, degrees of freedom and *P* value noted<br>*Give P values as exact values whenever suitable.*
☒	☐	For Bayesian analysis, information on the choice of priors and Markov chain Monte Carlo settings
☒	☐	For hierarchical and complex designs, identification of the appropriate level for tests and full reporting of outcomes
☒	☐	Estimates of effect sizes (e.g. Cohen's *d*, Pearson's *r*), indicating how they were calculated

Our web collection on statistics for biologists contains articles on many of the points above.

Software and code

Policy information about availability of computer code

Data collection	pClamp11.2, gromacs 2023, gromacs 5.0.4, plumed 2.1.3
Data analysis	RELION 5.0, cryoSPARC v4.4.1, coot 0.9.8.95, PHENIX 1.20, REFMAC5, Servalcat, UCSF Chimera 1.14, ChimeraX-1.8, Pymol 1.8.2.0, MolProbity v4.2, ProDy 2.5.0, Clampfit 11.2, GraphPad Prism 10.3.1

For manuscripts utilizing custom algorithms or software that are central to the research but not yet described in published literature, software must be made available to editors and reviewers. We strongly encourage code deposition in a community repository (e.g. GitHub). See the Nature Portfolio guidelines for submitting code & software for further information.

Data

Policy information about availability of data

All manuscripts must include a data availability statement. This statement should provide the following information, where applicable:

- Accession codes, unique identifiers, or web links for publicly available datasets
- A description of any restrictions on data availability
- For clinical datasets or third party data, please ensure that the statement adheres to our policy

Cryo-EM coordinates and corresponding EM maps are deposited in the PDB and EMDB under the following accession codes: Apo GluA3G439/γ2: 9HPD/EMD-52326 (LBD–TMD) and 9HPE/EMD-52327 (NTD-LBD); Active/open state GluA3G439/γ2: 9HPK/EMD-52332 (LBD–TMD) and 9HPC, EMD-52325 (NTD-LBD); Desensitized GluA3G439/γ2 NTD-LBD: 9HPF, EMD-52328; Apo GluA3G439,R163I/γ2 NTD-LBD: 9HPG/EMD-52329. Conventional MD and metadynamics simulations have been

deposited to the MDDB and accession codes will arrive following maintainer curation.

Source data for all electrophysiology experiments is also provided in a spreadsheet.

Research involving human participants, their data, or biological material

Policy information about studies with human participants or human data. See also policy information about sex, gender (identity/presentation), and sexual orientation and race, ethnicity and racism.

Reporting on sex and gender	N/A
Reporting on race, ethnicity, or other socially relevant groupings	N/A
Population characteristics	N/A
Recruitment	N/A
Ethics oversight	N/A

Note that full information on the approval of the study protocol must also be provided in the manuscript.

Field-specific reporting

Please select the one below that is the best fit for your research. If you are not sure, read the appropriate sections before making your selection.

☒ Life sciences ☐ Behavioural & social sciences ☐ Ecological, evolutionary & environmental sciences

For a reference copy of the document with all sections, see nature.com/documents/nr-reporting-summary-flat.pdf

Life sciences study design

All studies must disclose on these points even when the disclosure is negative.

Sample size	No statistical method was used to determine sample size. Cryo-EM sample sizes were determined by available electron microscopy time and the number of particles on electron microscopy grids. The sample size is sufficient to obtain a structure at the reported resolution, as assessed by Fourier shell correlation. Electrophysiology sample sizes were determined based on literature review, previous experience with data of this sort, and reproducibility of results across independent experiments. The authors have extensive previous experience with data of this type (Zhang, Nature 2021; Herguedas, Science 2019; Herguedas, Science 2016; Cais, Cell Reports 2014)., therefore sample sizes were based on understanding of sample variabilities.
Data exclusions	During cryo-EM data processing, data were excluded using standard classification approaches in cryoSPARC and RELION to remove false picks and particle images without high resolution content. In electrophysiology experiments, data were excluded based on pre-established quality control criteria (rise time, holding current).
Replication	All cryo-EM structures were determined from independent half datasets, which were compared to assess the resolution of the reconstruction. All electrophysiology data sets were pooled from at least two independent experiments and all results were successfully replicated.
Randomization	For Cryo-EM, division of datasets into two random halves was done based on standard approach in RELION. Randomization is not relevant to electrophysiology.
Blinding	Blinding was not applicable to cryo-EM or MD simulations, because this type of study does not use group allocation. Researchers were not blinded for the acquisition or analysis of electrophysiology data as it was not technically or practically feasible to do so. Experimenter independence was ensured by application of defined exclusion criteria as stated above.

Reporting for specific materials, systems and methods

We require information from authors about some types of materials, experimental systems and methods used in many studies. Here, indicate whether each material, system or method listed is relevant to your study. If you are not sure if a list item applies to your research, read the appropriate section before selecting a response.

Materials & experimental systems

n/a	Involved in the study	
☒	☐	Antibodies
☐	☒	Eukaryotic cell lines
☒	☐	Palaeontology and archaeology
☐	☒	Animals and other organisms
☒	☐	Clinical data
☒	☐	Dual use research of concern
☒	☐	Plants

Methods

n/a	Involved in the study	
☒	☐	ChIP-seq
☐	☒	Flow cytometry
☒	☐	MRI-based neuroimaging

Eukaryotic cell lines

Policy information about cell lines and Sex and Gender in Research

Cell line source(s)	HEK293Tcells were purchased from ATCC and HEK-Expi293F cells from ThermoFisher Scientific (Cat# A14527).
Authentication	No further authentication was performed for cell lines used in this study.
Mycoplasma contamination	No mycoplasma testing was performed specifically for this study, the HEK293T cell line had been tested negative in the past.
Commonly misidentified lines (See ICLAC register)	HEK cells are listed in the register; however, our HEK cell lines come from reliable source and are the only secondary cell type used in this study, which minimizes the risk of any cross-contamination.

Animals and other research organisms

Policy information about studies involving animals; ARRIVE guidelines recommended for reporting animal research, and Sex and Gender in Research

Laboratory animals	C57/Bl6 mice of both sexes were used in this study at age postnatal day 6-8. Animals were housed with unlimited access to food and water under a standard 12 hour light-dark cycle, at normal room temperature (approx 20-22 degrees Centigrade). Pregnant mothers were monitored daily, and P0 refers to the day of litter discovery.
Wild animals	No wild animals were used in this study.
Reporting on sex	Organotypic slices were prepared from pups of both sexes. There iare no reported or discernible differences between sexes in electrophysiological properties of slices prepared at age P6-8.
Field-collected samples	No field collected samples were used in this study.
Ethics oversight	All procedures were carried out under PPL PP5747704 in accordance with UK Home Office regulations. Experiments conducted in the UK are licensed under the UK Animals (Scientific Procedures) Act of 1986 following local ethical approval.

Note that full information on the approval of the study protocol must also be provided in the manuscript.

Plants

Seed stocks	N/A
Novel plant genotypes	N/A
Authentication	N/A

Flow Cytometry

Plots

Confirm that:

☒ The axis labels state the marker and fluorochrome used (e.g. CD4-FITC).

☒ The axis scales are clearly visible. Include numbers along axes only for bottom left plot of group (a 'group' is an analysis of identical markers).

☒ All plots are contour plots with outliers or pseudocolor plots.

☒ A numerical value for number of cells or percentage (with statistics) is provided.

Methodology

Sample preparation	Samples consisted of HEK293T cells transiently transfected with AMPAR subunits, with an HA tag inserted into the N-terminal domain following the signal peptide. Cells were harvested from 12-well plates using ice-cold PBS supplemented with 5% FBS, 1% BSA, and 0.05% sodium azide. Antibody incubation was performed in a final volume of 50 µL for 1 hour on ice. Following incubation, cells were washed three times with the same buffer and resuspended in 300 µL PBS containing 0.05% sodium azide immediately prior to analysis on an LSRFortessa flow cytometer. A minimum of 30,000 cells were collected per sample for analysis.
Instrument	BD LSRFortessa
Software	FlowJo (version 10.8.0).
Cell population abundance	A total of at least 30000 cells were taken for mean geometric fluorescence calculation
Gating strategy	Gating was used only to include the cells rather than debris. Further gating was not used.

☒ Tick this box to confirm that a figure exemplifying the gating strategy is provided in the Supplementary Information.
